# Improving Cull Cow Meat Quality Using Vacuum Impregnation

**DOI:** 10.3390/foods7050074

**Published:** 2018-05-07

**Authors:** Martha Y. Leal-Ramos, Alma D. Alarcón-Rojo, Néstor Gutiérrez-Méndez, Hugo Mújica-Paz, Felipe Rodríguez-Almeida, Armando Quintero-Ramos

**Affiliations:** 1 Facultad de Ciencias Químicas, Universidad Autónoma de Chihuahua, 31125 Chihuahua, Mexico; ngutierrez@uach.mx (N.G.-M.); aquinter@uach.mx (A.Q.-R.); 2 Facultad de Zootecnia y Ecología, Universidad Autónoma de Chihuahua, 33820 Chihuahua, Mexico; aalarcon@uach.mx (A.D.A.-R.); frodrigu@uach.mx (F.R.-A.); 3 Departamento de Biotecnología e Ingeniería de Alimentos, Instituto Tecnológico y de Estudios Superiores de Monterrey, 64849 Monterrey, Mexico; h.mujica@itesm.mx

**Keywords:** vacuum impregnation, sodium chloride brine, cull cows, meat quality, microstructure, moisture-enhanced meat

## Abstract

Boneless strip loins from mature cows (50 to 70 months of age) were vacuum impregnated (VI) with an isotonic solution (IS) of sodium chloride. This study sought to determine the vacuum impregnation and microstructural properties of meat from cull cows. The experiments were conducted by varying the pressure, $p_{1}$ (20.3, 71.1 kPa), and time, $t_{1}$ (0.5, 2.0, 4.0 h), of impregnation. After the VI step, the meat was kept for a time, $t_{2}$ (0.0, 0.5, 2.0, 4.0 h), in the IS under atmospheric pressure. The microstructural changes, impregnation, deformation, and porosity of the meat were measured in all the treatments. Impregnation and deformation levels in terms of volume fractions of the initial sample at the end of the vacuum step and the VI processes were calculated according to the mathematical model for deformation-relaxation and hydrodynamic mechanisms. Scanning electron microscopy (SEM) was used to study the microstructure of the vacuum-impregnated meat samples. Results showed that both the vacuum and atmospheric pressures generated a positive impregnation and deformation. The highest values of impregnation X (10.5%) and deformation γ (9.3%) were obtained at $p_{1}$ of 71.1 kPa and $t_{1}$ of 4.0 h. The sample effective porosity ($\varepsilon_{e}$) exhibited a significant interaction (*p* < 0.01) between $p_{1}\times t_{1}$. The highest $\varepsilon_{e}$ (14.0%) was achieved at $p_{1}$ of 20.3 kPa and $t_{1}$ of 4.0 h, whereas the most extended distension of meat fibers (98 μm) was observed at the highest levels of *p*_1_, *t*_1_, and *t*_2_. These results indicate that meat from mature cows can undergo a vacuum-wetting process successfully, with an IS of sodium chloride to improve its quality.

## 1. Introduction

In comparison to meat from young animals, beef from mature cows is usually tougher and less juicy [1]. As a result, 56.4% of mature cow meat is merchandised as beef trim for grinding and processing. The remaining 43.6% is sold at a lower price at the primal/subprimal level and as steaks/roasts in supermarkets and food service operations [2]. On the other hand, moisture enhancement is a value-adding meat processing technology widely used by the meat industry. This technology improves tenderness, juiciness, flavor, and consistency of whole-muscle meat products of reduced eating quality [3,4]. Moisture enhancement consists of adding a solution containing water, sodium chloride (NaCl), sodium phosphates, and sodium lactate as preservative into raw whole-muscle meat [3]. Salt and phosphates increase the water-holding capacity (WHC) of post-rigor meat by significantly altering its microstructure. Most of the water in muscle (95%) is held by capillary forces in the spaces arising from the arrangement of the thick filaments of myosin and the thin filaments of actin/tropomyosin within the myofibrils [5,6]. The introduction of salts into muscle tissue changes the number of charges on both thick and thin filaments, resulting in the repulsion and enlargement of the spaces between actin and myosin, which allows the incorporation of more water. When a greater amount of water is bound to the meat protein matrix, it becomes more swollen, softer, and juicier [5].

Moisture-enhanced meat (MEM) is prepared by injecting brine into sub-primal pieces (without further tumbling treatment), which are then portioned into steaks or small pieces of meat [3,4]. Unfortunately, current methods for brine injection in meat have significant disadvantages; even when brine is injected from multiple needles, as is common in commercial practice, the brine is not uniformly distributed throughout the meat. This uneven distribution of brine causes striping or streaking which can be seen as “light” and “dark” stripes over the MEM’s surface, negatively impacting its appearance and overall quality [7,8]. Several studies have investigated novel techniques to overcome these problems and to accelerate mass transfer in whole-muscle meat-brine systems. The methods studied include ultrasound [9,10,11,12], high pressure [13,14,15], and vacuum impregnation (VI). Techniques involving VI have been used to reduce salting time and to increase water retention of dry-cured ham [16], tasajo [17], poultry [18], and salmon [19,20].

Vacuum impregnation allows the direct insertion of an external solution into a product through its pores in a fast, controlled, and uniform way without destroying its original structure [21]. Vacuum impregnation is conducted using consecutive vacuum and atmospheric pressure stages, which allow the impregnating solution to fill the food pores through deformation-relaxation and hydrodynamic mechanisms (HDM) [22]. The effect of VI on physical and structural properties of fruits has been studied in several products through the introduction of hypotonic, isotonic, and hypertonic solutions in plant tissue [23]. In the case of meat tissue, salts (sodium chloride and phosphates) by themselves can affect the microstructure of meat, specifically, the size of spaces between myosin and actin myofilaments that constitute the capillary pores of the meat. Nevertheless, it has been reported that pork meat that was treated in a brine of 0.005 g/cm^3^ NaCl showed slight differences in comparison with the untreated sample, while the essential structure of the myofibrils appeared to be intact [24].

Factors affecting the effectiveness of VI on food matrices include the following: (1) flow properties of the external liquid; (2) operation variables such as compression ratio ($r\approx p_{2}/p_{1}$), where $p_{1}$ is the vacuum pressure applied to the solid food-liquid system, and $p_{2}$ is the atmospheric pressure, lengths of the vacuum ($t_{1}$), and atmospheric ($t_{2}$) pressure steps, temperature, etc.; and (3) mechanical and structural properties of the food solid matrix (porosity, pore size and shape, type of fluid-like gas or liquid occupying the pores, viscoelastic character, etc.) [25]. Vacuum impregnation properties of food are characteristic parameters which show the feasibility of liquid penetration during VI processes. The primary VI properties of a food including the following: sample volume fraction impregnated by the external liquid at the end of the vacuum step ($X_{1}$) and at the end of the VI process (X); sample volume deformation at the end of the vacuum step ($\gamma_{1}$) and at the end of the VI process (γ); the sample effective porosity ($\varepsilon_{e}$).

Currently, no information is available on the VI properties of meat from cull cows or its related microstructural changes. The aim of this work was to study the effects of the VI operation variables $p_{1}$, $t_{1}$, and $t_{2}$ on the sample impregnation and deformation levels at the end of the vacuum step ($X_{1}$ and $\gamma_{1}$) and at the end of the VI process (X and γ). It also aimed to examine VI effects on both sample effective porosity ($\varepsilon_{e}$) and related microstructural changes of meat from mature cows vacuum impregnated with an isotonic solution of sodium chloride to improve the quality of cull cow meat.

## 2. Materials and Methods

### 2.1. Raw Material and Sample Preparation

Boneless strip loins (IMPS #180) [26] of five cull cows (one loin from each cow) of varying breeds (Brangus, Charolais, Holstein, and unknown crossbreeds) and varying ages (approximately 50 to 70 months of age) were used. One loin from each Brangus, Charolais, and Holstein, and two loins from unknown crossbreds, respectively, were used. Loins were obtained from a Federal Inspection Type (TIF) slaughter plant, in Chihuahua, Chih., Mexico (28°38′07′′ N, 106°05′20′′ W), at 24 h after slaughter (refrigerated at 4 °C). The TIF plants are regulated based on the guidelines of the Mexican Ministry of Agriculture, Livestock, Rural Development, Fisheries and Food and meet the sanitary standards imposed by the United States Department of Agriculture (USDA). In the TIF abattoir, after being stunned by a captive bolt (model USSS-1 JARVIS^®^), the cattle were slaughtered, suspended by a hind leg, bled, and transferred to the production line to begin the process of removing the head, feet, skin, viscera, and the quartering of the carcass.

Loins were transported to the laboratory and stored at 2 °C for no more than five days. Beef strip loins were trimmed of excess fat and connective tissues. Each strip loin was cut into 50 sections (6 × 5 × 2 cm) with the slab’s length perpendicular to the main loin axis.

### 2.2. Isotonic Solution Preparation

An isotonic solution (IS) was used in the VI experiments to avoid cell turgor alterations in beef. The IS was formulated with distilled water and sodium chloride (analytical grade). Water activity ($a_{w}$) of the IS was adjusted to that of fresh meat using the Favetto and Chirife equation (Equation (1)) [22], where k was the sodium chloride constant (0.0371), and m was the sodium chloride concentration (mol/kg water). The water activity of IS was confirmed with a hygrometer (Novasina IC-500AW-Lab, Talstrasse, Switzerland).

(1) $$a_{w}=1-km$$

### 2.3. Analytical Methods

Representative samples taken from each loin were ground and homogenized separately. Homogenates were used for moisture, fat, protein, and ash content analysis (AOAC Official Methods no. 950.46, 991.36, 981.10, and 920.153, respectively [27]). Meat homogenates (1 g) were manually mixed with 9 mL of distilled water for 10 s. The pH of the mixtures was measured with a pH-meter (HANNA Instruments Model HI 255, Cluj-Napoca, Rumania) calibrated with standard buffer solutions at pH 4.0 and pH 7.0. Water activity ($a_{w}$) of fresh meat was determined with a hygrometer (Novasina IC-500AW-Lab, Talstrasse, Switzerland). The pycnometer method was used to determine the densities of IS ($\rho_{\mathrm{IS}}$) at 2 ± 1 °C and paraffin wax ($\rho_{\mathrm{wax}}$). All analyses were performed in triplicate.

For each section of meat (24 sections by each strip loin), the initial mass ($M_{0}$) was determined. Sample mass at the end of the vacuum step ($M_{1}$) or sample mass at the end of the VI process (M) was determined as appropriate. Initial volume ($V_{0}$) for each section of meat was measured using the buoyant force method based on Archimedes’ principle [28]. A vessel containing a specific volume of IS at 2 ± 1 °C was placed on the scale plate and tared to zero. After its initial weight determination, each section of fresh meat was individually suspended by a nylon thread (0.05 mm thickness and 15 cm length) and wholly immersed in the IS so that the sample did not touch the walls or the bottom of the container. The individual immersion of each meat sample was so rapid (less than 2 min on average), that no significant changes in the temperature of the IS were observed when measured with a T-type thermocouple thermometer (Hanna Instruments Model HI 935004P, Cluj-Napoca, Rumania). The mass of liquid displaced by the sample ($M_{B}$) was recorded. Diameter and mass values of nylon thread were much smaller than those of meat sample; for this reason, these were neglected. The initial volume and apparent density of fresh meat $\rho_{\mathrm{meat}}$ (the density of meat including all remaining pores) were calculated by Equations (2) and (3), respectively.

(2) $$V_{0}=\frac{M_{B}}{\rho_{\mathrm{IS}}}$$

(3) $$\rho_{\mathrm{meat}}=\rho_{\mathrm{IS}}\cdot \frac{M_{0}}{M_{B}^{\prime }}$$

For measuring sample volume at the end of the vacuum step ($V_{1}$), each section of meat was coated with a thin layer of paraffin wax. The wax layer avoided the mass transfer between sample and liquid. Each wax-coated sample was weighed and wholly immersed again in the IS as mentioned previously. The mass of liquid displaced by the wax-coated sample ($M_{B}^{\prime }$) was recorded. The mass of the wax layer ($M_{\mathrm{wax}}$) was obtained by subtracting $M_{1}$ from the mass of the wax-covered meat sample. Sample volume at the end of the vacuum step was calculated by Equation (4).

(4) $$V_{1}=\frac{M_{B}^{\prime }}{\rho_{\mathrm{IS}}}-\frac{M_{\mathrm{wax}}}{\rho_{\mathrm{wax}}}$$

To measure the sample volume at the end of the vacuum impregnation process (V), each sample was coated with a thin layer of paraffin wax and treated like the sample at the end of the vacuum step. Five sections of meat by treatment were used.

### 2.4. Vacuum Impregnation Experiments

Vacuum impregnation treatments were carried out in a vacuum system. The vacuum system comprised a glass vacuum desiccator (Wheaton Dry-Seal, capacity 5 L) containing IS at 2 ± 1 °C and a vacuum pump (Felisa pump, Mod. 1600 L, México), both connected through a silicone vacuum hose. From a beef strip loin, 24 sections of meat were taken and assigned to six desiccators (four pieces per desiccator). Meat sections were immersed in the IS per the ratio meat: IS of 1:5 (*w*/*w*). The vacuum pressure $p_{1}$ (20.3 or 71.1 kPa) was applied in the desiccator for a length of vacuum step $t_{1}$ (0.5, 2.0 or 4.0 h). Vacuum pressures used in this work were in the maximum and minimum range reported by others for similar materials [18]. At the end of the first vacuum step, atmospheric pressure ($p_{2}$) was restored while samples remained immersed for a time $t_{2}$ (0.0, 0.5, 2.0, or 4.0 h). For each meat section, the impregnation $X_{1}$ or X (m^3^ IS/m^3^ initial sample) and deformation $\gamma_{1}$ or γ (m^3^/m^3^ initial sample) levels in terms of volume fractions of the initial sample (Equations (5)–(9)) at the end of vacuum step and VI processes were calculated. It should be noted that the X values represent the actual impregnation given by $X_{1}+X_{2}$, where $X_{2}$ is the sample volume fraction impregnated by the external liquid during the atmospheric pressure step. Likewise, γ values represent the actual deformation given by $\gamma_{1}+\gamma_{2}$, where $\gamma_{2}$ is the sample volume deformed during the atmospheric pressure step.

For each meat section that was vacuum-impregnated, the effective or initial porosity ($\varepsilon_{e}$) was calculated according to the mathematical model (Equation (9)) reported by (Fito et al., 1996) [29], using these parameters and a compression ratio of $r\approx p_{2}/p_{1\mathrm{abs}}$ that considers the absolute vacuum pressure as $p_{1\mathrm{abs}}=p_{2}-p_{1}$. In these equations $\left(M_{1}-M_{0}\right)$ and $\left(M-M_{0}\right)$ represented the masses of IS impregnated in the samples at the end of the vacuum step and the end of the VI process, respectively.

(5) $$X_{1}=\frac{M_{1}-M_{0}}{\rho_{\mathrm{IS}}V_{0}}$$

(6) $$\gamma_{1}=\frac{V_{1}-V_{0}}{V_{0}}$$

(7) $$X=\frac{M-M_{0}}{\rho_{\mathrm{IS}}V_{0}}$$

(8) $$\gamma =\frac{V-V_{0}}{V_{0}}$$

(9) $$\varepsilon_{e}=\frac{\left(X-\gamma \right)r+\gamma_{1}}{r-1}$$

### 2.5. Scanning Electron Microscopy

Scanning electron microscopy (SEM) was used to study the microstructure of the vacuum-impregnated meat samples. Preparation and analysis of meat samples for SEM was conducted using the method proposed by Dykstra [30]. Meat samples (fresh and vacuum-impregnated) of approximately 2 mm^3^ were cut with a stainless steel surgical scalpel blade. Samples were fixed in 2.5% glutaraldehyde in 0.1 M (pH 7.3) sodium cacodylate buffer for 12 h at 4 °C. Afterward, samples were washed twice in 0.1 M sodium cacodylate (pH 7.3). Samples were then dehydrated in a graded series of 55, 75, 85, 95, and 100% ethanol (ethanol:water, *v*/*v*). Samples were placed in each dilution for 20 min. The final absolute ethanol treatment was repeated two times each for 20 min. The samples were ultra-dehydrated by the critical point with CO_2_ (1100 psi, 31.1 °C) in a Samdri-780A, Tousimis Research Corporation critical point dryer (Rockville, MD, USA). The dry samples were then mounted on aluminum stubs with carbon conductive cement, coated with gold, and observed in a JEOL JSM-5800LV (JEOL, Freising, Germany) scanning electron microscope operating at an accelerating voltage of 10 kV.

### 2.6. Statistical Analysis

The experimental design was a randomized complete block in a 2 × 3 × 4 factorial arrangement of treatments: two vacuum pressures $p_{1}$ (20.3 and 71.1 kPa) × three vacuum step lengths $t_{1}$ (0.5, 2.0 and 4.0 h) × four atmospheric step lengths $t_{2}$ (0.0, 0.5, 2.0, and 4.0 h). Each beef strip loin from cull cows represented a block for a total of five blocks. The mixed procedure of SAS (SAS^TM^ version 9.2) was used to perform type III tests of fixed effects. The model used for the sample impregnation (X), deformation (γ), and $\varepsilon_{e}$ data included $p_{1}$, $t_{1}$, $t_{2}$ and their interactions as fixed effects and block as a random effect. To test the trends in the effects of the lengths of the vacuum ($t_{1}$) and atmospheric ($t_{2}$) pressure steps when they were significant either as interactions with $p_{1}$ or as main effects without interaction effect, these effects were fitted as continuous rather than qualifying factors. First and second order regression coefficients were used for $t_{1}$ and first, second, and third order regression coefficients for $t_{2}$, under the complete model previously described and adjusted with a mixed procedure of SAS^TM^. Regression coefficients of the highest order that were not significant were eliminated from the model.

## 3. Results and Discussion

### 3.1. Analytical Results

The proximate composition, pH, water activity, and apparent density values of fresh meat from cull cows are presented in Table 1. Moisture, protein, fat, ash, and pH of the meat were similar to those reported by Buford et al. [31] and Patten et al. [32] for meat from mature cows. Water activity of fresh meat from cull cows (0.98) was similar to that reported for beef (0.98 to 0.99) by Lewicki [33]. Meat pH ranged from 5.62 to 5.97. It has been widely reported that muscle pH is dependent on the amount of glycogen present in the muscles at the time of slaughter. Muscle glycogen content may be influenced by the animal’s diet and stress before slaughter. If glycogen stores are depleted before slaughter, the pH will decline slowly, and a higher than normal ultimate pH will occur [32]. The amount of water bound within the muscle tissue greatly depends on the space available between the myofilaments of actin and myosin. This space forms the capillary pores of muscle tissue in which the pH value plays a vital role [3]. The highest water-holding capacity of fresh meat occurs near the isoelectric point of main meat proteins at pH 5.2 or in a range between 5.5 to 5.9 [3].

The apparent density value of meat from cull cows was 1.07 ± 0.01 g/cm^3^, whereas density values of IS and paraffin wax were 1.02 and 0.76 g/cm^3^, respectively (Table 1). Hildrum et al. [34] stated that lean meat tissue has a consistent density of 1.07 to 1.08 g/cm^3^. Cull cows are variable with regard to breed, size, age (from first calving dairy cows, culled for low production, to mature cows), fatness, and physiological and health status [35]. Consequently, as a result of their heterogeneity, considerable variability exists in the chemical composition, structural-mechanical properties, and quality of this meat.

### 3.2. Volume of Brine Impregnated in the Meat with Vacuum Impregnation

**Figure 1:**
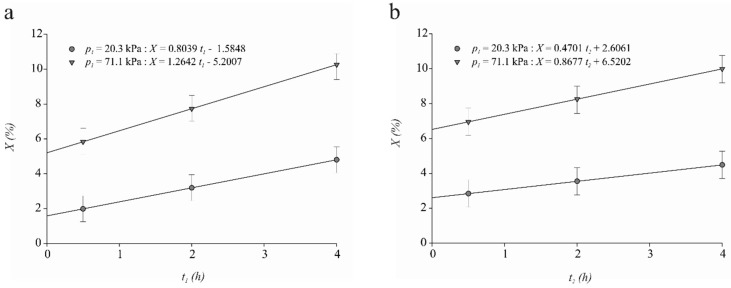
Mean ± Standard error (SE, *n* = 120) for meat sample volume fraction vacuum impregnated by the isotonic sodium chloride solution (X) for both levels of vacuum pressure ($p_{1}$) (mean ± SE): (**a**) as a function of the length of the vacuum pressure step ($t_{1}$) and, (**b**) as a function of the length of the atmospheric pressure step ($t_{2}$).

The sample volume fraction impregnated by the IS at the end of the VI process (X) as a function of the time at which the system was under vacuum ($t_{1}$) and under atmospheric pressure ($t_{2}$) for both levels of vacuum pressure ($p_{1}$) are shown in Figure 1a,b, respectively. Part of X was impregnated during $t_{1},$ which corresponds to $X_{1}$ (Figure 1a). In all cases, positive values of $X_{1}$ were achieved. In the vacuum step, two opposite fluxes occurred in the pores of a food: (1) the expansion and partial outflow of the product’s internal gas which was accompanied by the product pore’s native liquid and (2) the capillary inflow of the external liquid as a function of the interfacial tension of the liquid and the diameter of pores [23,29,36]. An important part of the pores in the matrix of meat is occupied by a free liquid phase that contains a small or neglected gas phase volume entrapped within it [25]. This free liquid phase may be released from the matrix in the vacuum step. Chiralt et al. [25] demonstrated that, in Manchego curd (cylinders) with low porosity (like meat), the amount of liquid phase that can be released by applying vacuum (50 mbar) was 2.3% and 3.4% (expressed as curd volume fraction), respectively, before and after submitting curd to a VI operation with an isotonic solution. This low porosity causes approximately 3% of the curd volume to be replaced by brine in the brine vacuum impregnation process, although the curd volume fraction occupied by the gas is minute (less than 1%). Positive values of $X_{1}$ could indicate that the capillary solution gain of the meat samples at vacuum pressure dominated over the losses of native liquid.

**Figure 2:**
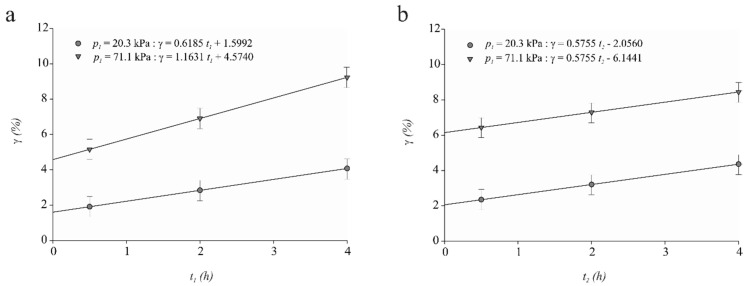
Mean ± SE (*n* = 120) for final meat sample volume deformation (γ) for both levels of vacuum pressure ($p_{1}$): (**a**) as a function of the length of the vacuum pressure step ($t_{1}$) and (**b**) as a function of the length of the atmospheric pressure step ($t_{2}$).

Salvatori et al. [21] reported a positive value of $X_{1}$ (0.9%) for mangoes, which was related to the capillary penetration of an external solution due to its fibrous structure. Both meat and mangoes have fibrous structures. Fito and Chiralt [23] reported that, if during VI process the $p_{1}$ > 200 mbar (20 kPa), then a great capillary contribution can be expected for products with a small pore diameter (≈10 μm or less). The spaces between the thick filaments of myosin and the thin filaments of actin-tropomyosin within myofibrils constitute the capillary pores where most of the water in meat is present [37]. Interfilament space has been observed to vary between 320 to 570 Å (0.03 to 0.06 μm) with pH, sarcomere length, ionic strength, osmotic pressure, and whether the muscle is pre- or post-rigor [37]. In viscoelastic matrices such as meat, pressure changes can promote sample deformations coupled with impregnation in the vacuum step where sample expansion occurs as well as in the atmospheric pressure step or compression period [25]. Values of sample volume fraction impregnated by the solution at the end of the vacuum step (Figure 1a) and at the end of the atmospheric pressure step (Figure 1b) are similar to the values of relative sample volume deformation at the end of the vacuum period (Figure 2a) as well as at the end of the atmospheric pressure period (Figure 2b). The above results indicate that deformation-relaxation phenomena dominate the hydrodynamic mechanism.

Meat sample volume fraction impregnated by the IS at the end of the VI process (X) exhibited significant (*p* < 0.01) $p_{1}\times t_{1}$ and $p_{1}\times t_{2}$ interaction effects, but no other interaction effects were important (*p* < 0.05). Figure 1a showed that the greater the vacuum level applied in the first step and the longer the vacuum step, the greater the average final impregnation degree (*p* < 0.01). The X was increased on average 0.80% ± 0.15 (*p* < 0.01) and 1.26% ± 0.15 (*p* < 0.01) by each hour that the system was under a $p_{1}$ of 20.3 kPa and 71.1 kPa, respectively (Figure 1a). Therefore, the highest average value of impregnation (10.4%) reached during the vacuum step was obtained at $p_{1}$ of 71.1 kPa applied by $t_{1}$ of 4.0 h (Figure 1a).

This observed phenomenon may have been a result of the high vacuum level, which allowed for better removal of native liquid from the meat pores. It also may also have resulted in a decrease in the remaining native liquid volume, with the same volume of external liquid, which was penetrated by HDM, as an effect of capillary pressure. Chiralt et al. [25] reported that the higher vacuum level $p_{1}$ in the system, the greater the capillary penetration in meat, fish, and cheese vacuum impregnated with a sodium chloride solution. On the other hand, the longer the vacuum step, the greater amount of native liquid left the meat structure, resulting in greater penetration of the external liquid.

Figure 1b shows that the average rates of change of X with respect to $t_{2}$ were 0.47% ± 0.17 (*p* < 0.01) and 0.87% ± 0.17 (*p* < 0.01) at 20.3 and 71.1 kPa, respectively (*p* < 0.01). It is worth mentioning that part of X impregnated during $t_{2}$ corresponds to $X_{2}$. Therefore, the highest average value of $X_{2}$ (9.7%) was reached at $t_{2}$ of 4.0 h, when the vacuum pressure applied to the system was 71.1 kPa (Figure 1b). When external pressure was restituted in the system, greatly deformed matrices may have relaxed the mechanical energy stored in their elastic structural elements which was in line with a continuous impregnation of their pores. The mechanical relaxation level and subsequent sample volume recovery depended on the viscoelastic properties of the matrix; the greater the elastic character, the higher the volume recovery and the coupled impregnation.

Brines for 10%-injected meat contain approximately 3% phosphates, 6% salt, and 30% lactate [3]. Notably, the average volume of the isotonic sodium chloride solution which was vacuum-impregnated in the meat during the vacuum step (10.4%) was very similar to that for injected meat (10%). Thus, the coupling of the hydrodynamic mechanism with deformation-relaxation phenomena produced a weight gain in each piece of meat without using high levels of either salt or sodium-based phosphates and lactate. Intake of dietary sodium has been linked to hypertension and, consequently, an increased risk of cardiovascular disease. In developed countries, meat and meat products contribute 21% to the sodium intake [38]. A challenge for the meat industry is to produce reduced-sodium raw or cooked meat products that consumers can enjoy as part of an ongoing healthier diet and lifestyle. Vacuum impregnation could be an excellent alternative to achieve moisture-enhanced meat products with a similar weight gain to traditional injected meats but with lower levels of sodium.

### 3.3. Volume Deformation of Cull Cow Meat Subjected to Vacuum Impregnation

The sample volume deformation at the end of the VI process (γ) as a function of the lengths of the vacuum ($t_{1}$) and atmospheric ($t_{2}$) pressure steps for both levels of vacuum pressure ($p_{1}$) are shown in Figure 2a,b, respectively. Meat samples increased in volume during the vacuum pressure step, as evidenced by positive values of $\gamma_{1}$(Figure 2a). This change in volume could be related to the prevailing capillary penetration of the external liquid during the vacuum step which promoted distension of the solid matrix of meat. Meat sample volume deformation at the end of the VI process (γ) exhibited a significant (*p* < 0.01) $p_{1}\times t_{1}$ interaction.

The γ was increased on average 0.62% ± 0.14 (*p* < 0.01) and 1.16% ± 0.13 (*p* < 0.01) by each hour that the system was under a $p_{1}$ of 20.3 kPa and 71.1 kPa, respectively (*p* < 0.01) (Figure 2a). The highest mean value of γ (9.3%) was obtained at $p_{1}$ of 71.1 kPa and $t_{1}$ for 4.0 h. This change could be explained by the fact that the application of a more intense and prolonged vacuum step during the vacuum impregnation process may lead to a better substitution of a part of the sample-free liquid phase by the external liquid. This, in turn, will assist in the overall increase of sample volume. On the other hand, the length of the atmospheric step ($t_{2}$) had a significant positive linear effect (*p* < 0.01) on γ for both levels of $p_{1}$ (Figure 2b). This could have been a result of the water added during the atmospheric step, which allowed for an increase in the overall volume of the meat sample.

Other factors affecting the sample volume deformation at the end of the vacuum impregnation process were the mechanical properties of the food. Chiralt et al. [25] stated that the mechanical relaxation level and subsequent sample volume recovery would depend on the viscoelastic properties of the food matrix; the greater the elastic character, the higher the volume recovery and coupled impregnation. The connective tissue of meat from old cull cows is characterized by a higher number of cross-links within the collagen molecules. Collagen, the main component of muscle connective tissue, greatly influences the mechanical properties of meat. Lepetit [39] stated that the elastic modulus of the collagenous fraction of connective tissues is approximately proportional to the total number of collagen cross-links present per volume of meat. Thus, the greatest elastic character of meat from older cull cows could explain the positive values of γ observed.

### 3.4. Changes in the Sample Effective Porosity of Meat

**Figure 3:**
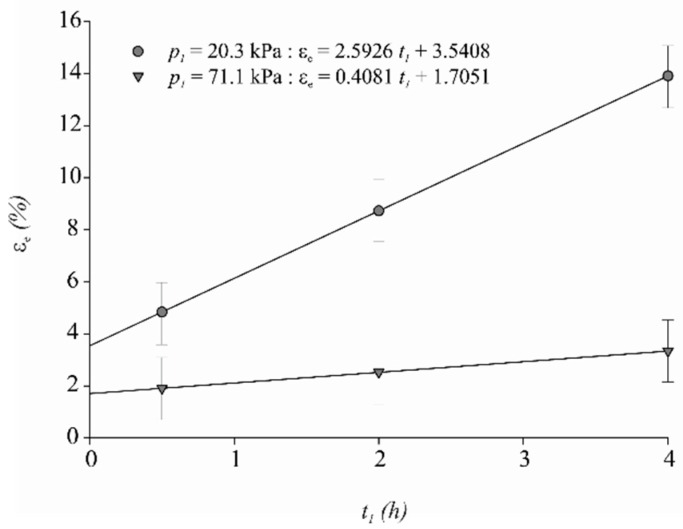
Mean ± SE (*n* = 120) for meat sample effective porosity ($\varepsilon_{e}$) as a function of the length of the vacuum step ($t_{1}$) for both levels of vacuum pressure ($p_{1}$).

The effective porosity ($\varepsilon_{e}$) of the meat sample as a function of $t_{1}$ exhibited a significant $p_{1}\times t_{1}$ interaction effect (*p* < 0.01), as shown Figure 3. The $\varepsilon_{e}$ increased 2.59% ± 0.25 (*p* < 0.01) and 0.41% ± 0.23 (*p* < 0.01) at 20.3 and 71.1 kPa, respectively (Figure 3). The $t_{2}$ did not have a significant effect (*p* > 0.98) on $\varepsilon_{e}$. The highest mean value of $\varepsilon_{e}$ (14.0%) was obtained at $p_{1}$ for 20.3 kPa and $t_{1}$ of 4.0 h. Effective porosity is expressed a priori as the percentage of sample volume initially occupied by the gases but is defined more precisely as the sample volume fraction available for the deformation-relaxation phenomena, coupled with the hydrodynamic mechanism as indicated in Equation (9) [40]. Thus, 14.0% of the meat sample volume was occupied by pores or capillaries that could be impregnated with an external solution. The capillaries consist of the spaces between the actin and myosin filaments. It has been established that a high vacuum level increases the porosity of most plant tissues as a result of a high expansion and release of the gas inside the pores of vegetables. Moreover, a high vacuum level allows for the better removal of native liquid from the tissue structure. In this way, after the restoration of atmospheric pressure, a greater volume is available for impregnation phenomena [41]. In this study, it was observed that the meat sample effective porosity behaved contrary to that observed in vegetable tissues. The highest value of $p_{1}$ applied for longer $t_{1}$ promoted a greater impregnation level at the end of vacuum pressure step by the capillary. When the atmospheric pressure was restored in the meat–IS system, a reduced volume was available for the impregnation by HDM, which was observed by smaller values of $\varepsilon_{e}$.

### 3.5. Microstructural Analysis of Meat Vacuum Impregnated

**Figure 4:**
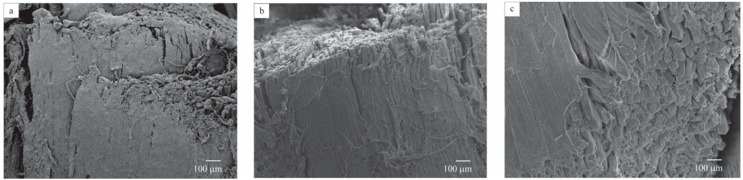
Scanning electron microscopy (SEM) micrographs of (**a**) fresh meat from cull cows; (**b**) vacuum-impregnated meat at $p_{1}$ of 20.3 kPa, $t_{1}$ of 0.5 h, and $t_{2}$ of 0.5 h, and (**c**) vacuum-impregnated meat at $p_{1}$ of 71.1 kPa, $t_{1}$ of 4.0 h, and $t_{2}$ of 4.0 h at 80× magnification.

The effect of vacuum impregnation on the microstructure of meat from cull cows is shown in Figure 4. As can be seen, meat sample vacuum impregnated at $p_{1}$ of 20.3 kPa, $t_{1}$ of 0.5 h and $t_{2}$ of 0.5 h (Figure 4b) showed slight swelling (approximately 50 μm) of muscle fibers with respect to that of fresh meat, whose essential structure of myofibrils appeared to remain intact (Figure 4a). Swelling of muscle fibers was more pronounced in the case of the vacuum-impregnated meat at the highest levels of factors studied. The largest thickness (98 μm) of muscle fibers was achieved at $p_{1}$ of 71.1 kPa, $t_{1}$ of 4.0 h, and $t_{2}$ of 4.0 h (Figure 4c). These results confirm the findings previously described with regard to the deformation properties of meat (Section 3.2). The influence of VI on meat microstructure could be explained by a more complete and homogeneous interchange of the gas and native liquid occupying the open pores (intermyofibrillar spaces) of the meat by the external isotonic sodium chloride solution. This results in a more complete contact between sodium chloride and meat proteins which leads to a more pronounced swelling of the meat matrix.

Graiver et al. [24] found that pork meat treated in a brine of 5 g/L NaCl showed slight differences to an untreated sample; however, the essential structure of the myofibrils appeared to remain intact. Fibers immersed in a NaCl solution of 140 g/L showed swelling as a result of salt ions diminishing the attractive forces between adjacent protein molecules. This resulted in an enlargement of the distance between the proteins and the meat which underwent swelling. Meat treated in a brine of NaCl 330 g/L produced fragmented and dehydrated fibers with a granular appearance. In the present study, 18.95 g/L brine concentration was applied. Therefore, one may assume that microstructure changes were predominantly caused by the vacuum impregnation treatment as opposed to the effects of salt.

## 4. Conclusions

For all vacuum impregnation conditions studied, positive values of impregnation ($X_{1}$ and X) and deformation ($\gamma_{1}$ and γ) were obtained. The coupling of a hydrodynamic mechanism with deformation-relaxation phenomena during a vacuum impregnation process with an isotonic sodium chloride solution resulted in a brine gain in each piece of meat. This brine gain was similar to the most common level of injected solution in moisture-enhanced meat (10%) without using brine injection or high levels of salt and sodium phosphates which increase sodium levels in meat. Contrary to processes in many plant tissues, the highest value of $p_{1}$ applied for longer $t_{1}$ promoted a greater impregnation level at the end of vacuum pressure step by capillary. This resulted in a diminished volume of meat samples available for impregnation by HDM when atmospheric pressure was restored in the meat–IS system. Accordingly, there were smaller values of $\varepsilon_{e}$ as $p_{1}$ increased and $t_{1}$ decreased.

Vacuum impregnation was proven to be a useful technique in the addition of an external solution to small pieces of meat from cull cows. Vacuum impregnation may present a good alternative in the production of moisture-enhanced meat products, offering similar weight gain as that of traditional injected meat but with lower levels of sodium and a more complete distribution of the brine into meat. The amount and distribution of water inside raw meat has a considerable influence on its tenderness and juiciness after cooking. A high amount of intra-myofibrillar water is associated with more tender meat.

## Figures and Tables

**Table 1:** Physical-Chemical Characteristics of Meat, Isotonic Solution and Paraffin Wax.

*Properties*	*Mean* ± SD
Meat	
Moisture (g/100 g meat) *	75.5 ± 3.0
Protein (g/100 g meat) *	20.2 ± 0.4
Fat (g/100 g meat) *	3.5 ± 2.5
Ash (g/100 g meat) *	1.2 ± 0.0
pH	5.8 ± 0.2
Water activity	0.98 ± 0.0
$\rho_{\mathrm{meat}}$(g/cm^3^)	1.07 ± 0.0
Isotonic solution	
Water activity	0.98 ± 0.0
$\rho_{\mathrm{IS}}$(g/cm^3^)	1.02 ± 0.01
Paraffin wax	
$\rho_{\mathrm{wax}}$(g/cm^3^)	0.76 ± 0.01

SD: standard deviation; *: values on wet weight basis.
